# In Vitro Evidence of Statins’ Protective Role against COVID-19 Hallmarks *Comptes* Rendus

**DOI:** 10.3390/biomedicines10092123

**Published:** 2022-08-29

**Authors:** Donatella Fiore, Maria Chiara Proto, Silvia Franceschelli, Maria Pascale, Maurizio Bifulco, Patrizia Gazzerro

**Affiliations:** 1 Department of Pharmacy, University of Salerno, 84084 Fisciano, SA, Italy; 2 Department of Molecular Medicine and Medical Biotechnologies, University of Naples “Federico II”, 80131 Naples, NA, Italy

**Keywords:** SARS-CoV-2, statins, COVID-19, cytokine storm, ACE2 receptor, lipid rafts

## Abstract

Despite the progressions in COVID-19 understanding, the optimization of patient-specific therapies remains a challenge. Statins, the most widely prescribed lipid-lowering drugs, received considerable attention due to their pleiotropic effects, encompassing lipid metabolism control and immunomodulatory and anti-thrombotic effects. In COVID-19 patients, statins improve clinical outcomes, reducing Intensive Care Unit admission, the onset of ARDS, and in-hospital death. However, the safety of statins in COVID-19 patients has been debated, mainly for statins’ ability to induce the expression of the ACE2 receptor, the main entry route of SARS-CoV-2. Unfortunately, the dynamic of statins’ mechanism in COVID-19 disease and prevention remains elusive. Using different in vitro models expressing different levels of ACE2 receptor, we investigated the role of lipophilic and hydrophilic statins on ACE2 receptor expression and subcellular localization. We demonstrated that the statin-mediated increase of ACE2 receptor expression does not necessarily coincide with its localization in lipid rafts domains, particularly after treatments with the lipophilic atorvastatin that disrupt lipid rafts’ integrity. Through a proteomic array, we analyzed the cytokine patterns demonstrating that statins inhibit the release of cytokines and factors involved in mild to severe COVID-19 cases. The results obtained provide additional information to dissect the mechanism underlying the protective effects of statin use in COVID-19.

## 1. Introduction

Despite the progress in understanding the 2019 coronavirus disease (COVID-19) and the development of efficacious vaccines, after two years the large-scale infection, the rapid spread, and the continued evolution of SARS-CoV-2 raise the urgent need to design a guideline for the management of COVID-19 patients.

Clinically, COVID-19 is highly variable, commonly ranging from an asymptomatic to a mild course. However, in a significant percentage of patients, the disease has a severe course that progresses to acute respiratory distress syndrome (ARDS) and multiple organ dysfunction syndrome, due to an alteration of the inflammatory and immune response, known as “cytokine storm” [1]. To enter the host cells, SARS-CoV-2 recognizes ACE2 (Angiotensin-converting enzyme 2) receptor and, subsequently, the serine protease TMPRSS2 is involved in the S protein priming [2]. ACE2 is localized in lipid rafts, small membrane microdomains enriched in cholesterol and glycosphingolipids, which are fundamental components in the entry and pathogenesis of the coronaviruses [3]. ACE2 receptor is expressed in different tissues and organs including colon, heart, brain, kidneys, and lungs, and its wide distribution could explain the multi-organ failure triggered by SARS-CoV-2 infection [1]. Once internalized through a mechanism that involves lipid rafts-dependent endocytosis [3], SARS-CoV-2 triggers the simultaneous activation of multiple receptors that culminates in uncontrolled and generalized inflammatory response, with an abnormal recruitment of immune cells and production of cytotoxic molecules. The overblown production of pro-inflammatory mediators, amplifying the inflammation process, translates into serious tissues damage, ARDS, and cardiovascular complications [1].

Since the beginning, the clinical profile of COVID-19 patients has been finely characterized, aiming to identify diagnostic and prognostic factors. The peculiar cytokine storm syndrome implies the production of several molecules (cytokines, chemokines, and other factors) specifically associated with SARS-CoV-2 infection and disease phase. COVID-19 is associated with abnormal levels of pro-inflammatory cytokines and chemokines such as IFN-γ, TNF-α, TGF, IL1, IL2, IL6, IL10, IL12, M-CSF, G-CSF, GM-CSF, IP-10, MCP-1, MIP 1, vascular endothelial growth factor (VEGF), RANTES, and many others. Interestingly, stage-specific patterns of these factors have been characterized [4,5,6].

In the acute phase of severe COVID-19, the main cardiovascular complications are myocarditis, myocardial injury and infarction, heart failure, acute coronary syndrome, pulmonary hypertension, right ventricular dysfunction, and arrhythmia [7,8]. Of note, increasing evidence highlighted the incidence of cardiovascular complications as a long-term consequence of COVID-19 [9]. On the other hand, in severe disease the cardiovascular manifestations are often a product of the worsening of previously existing conditions. It is indeed widely accepted that several comorbidities constitute a prediction factor for disease severity, which define the frail category. Beyond age, the majority of severe to mild COVID-19 patients have underlying diabetes, hypertension, hyperlipidemia, obesity, or atherosclerotic cardiovascular disease that predispose to worsen prognosis [10]. This evidence suggest that dysregulations of lipid metabolism and homeostasis play a central role in COVID-19 that cannot be overlooked.

The pandemic challenge has raised the urgency of rapid and cost-effective therapeutic strategies to counteract the spread of SARS-CoV-2 infection. Among others, the role of statins has been widely considered, even if debated, in COVID-19 [11,12,13,14]. Statins are 3-hydroxy-3-methyl-glutaryl-coenzyme A (HMG-CoA) reductase inhibitors and the most widely used lipid-lowering drugs in cardiovascular disease prevention. The interest in statins resides in their double effect in lipid metabolism control and immunomodulatory, anti-inflammatory, and anti-thrombotic effects, all allied effects in counteracting SARS-CoV-2 infection [13,15]. Previous observation suggested statins as potential anti-viral drugs in several infectious diseases such as Ebola (EBOV), Zika (ZKV), Influenza virus, HCV, HIV, and others [13].

As mevalonate pathway inhibitors, statins inhibit cholesterol and isoprenoid intermediate synthesis, thus potentially interfering in several steps of virus life cycle, such as entry through the plasma membrane, replication, virion maturation, and release [13]. Despite this, in the early phase of the SARS-CoV-2 pandemic, many doubts arose about the safety of statin use in COVID-19 due to their effect on ACE2 upregulation and the fear of an increased risk of infectiveness. However, paradoxically, statins counteract SARS-CoV-2 infection because the increase of the ACE2 receptor is required for the conversion of the pro-inflammatory Angiotensin 2 (AT2) in AT1-7, finally mitigating inflammation and tissue damage [13,16,17]. In fact, several clinical studies highlighted the safety of statins in COVID-19 cases and their efficacy in reducing the severe disease and often in lowering mortality [18,19,20,21]. In addition, statin discontinuation is associated with an increased risk of mortality in COVID-19 patients [22].

Recently, it has been demonstrated that atorvastatin inhibits SARS-CoV-2 D614G, Delta, and Mu variants, in vitro. In an in silico analysis, atorvastatin has shown a favorable binding affinity with SARS-CoV-2 RdRp and 3CL protease [23]. Similarly, simvastatin limits SARS-CoV-2 replication and mitigates inflammatory response both in vitro and in vivo. Interestingly, this mechanism seems related to simvastatin-mediated displacement of ACE2 receptor from lipid rafts [24]. Zapatero-Belinchón and colleagues demonstrated that Fluvastatin inhibits SARS-CoV-2 infection in vitro and ex vivo, modulating the expression of targets involved in protein translation and replication of the virus, but without affecting host innate immune response [25].

Overall, statins could represent a potentially valid tool to be used in conjunction with therapeutic protocols currently in clinical use. The statins also have the advantage of being drugs which have been extensively tested, well tolerated, and possessing a good pharmacological profile, potentially useful in improving the prognosis of SARS-CoV-2 infection, especially in frail patients. However, the full understanding of their beneficial molecular mechanisms in COVID-19 is still a challenge.

Here we investigated the hypothesis that the statin-mediated lipid perturbation could play a fundamental role in regulating host cell response at multiple levels, including receptor expression and localization, as well as release of damage promoting tissue factors and chemokines, aimed to dissect the dynamic of statins’ mechanisms in COVID-19 disease and prevention, also paying attention to different effects of lipophilic and hydrophilic statins.

## 2. Materials and Methods

### 2.1. Cell Cultures

The human A549-hACE2 cells, stably transfected to express the human *ACE2* gene, were obtained from InvivoGen (San Diego, CA, USA) and cultured in Dulbecco’s modified eagle medium (DMEM) with 4.5 g/L glucose, 10% of heat-inactivated FBS (Fetal Bovine Serum), 2 mM L-glutamine, 100 U/mL Pen-Strep, and 10 mg/mL of Puromycin as selection antibiotic. Human CaCO2 cells were purchased from ATCC (American Type Culture Collection) and cultured in Eagle’s Minimal Essential Medium (EMEM) with 20% FBS, 2 mM L-glutamine, 100 U/mL Pen-Strep, and 1% non-essential amino acids. Normal Human lung fibroblast cells MRC5 were purchased from ATCC and cultured in DMEM with 4.5 g/L glucose, 10% of heat-inactivated FBS (Fetal Bovine Serum), 2 mM L-glutamine, and 100 U/mL Pen-Strep. All the cell lines were routinely grown in monolayers and maintained at 37 °C in 5% CO_2_-humified atmosphere and regularly tested for mycoplasma presence.

### 2.2. Reagents, Treatments, and Antibodies

Atorvastatin (ATS) and Pravastatin (PVS), lipophilic and hydrophilic, respectively, were purchased from Selleckchem and dissolved in sterile DMSO. LPS (Lipopolysaccharides) was purchased from Sigma–Aldrich (St. Louis, MO, USA).

To obtain an adequate inflammatory stimulus, cells were treated for 48 h with ATS or PVS and subsequently stimulated with LPS 1 µg/mL for 4 h before the end of treatment in A549-hACE2 and MRC5 cells, or for 24 h in CaCO2 cells.

Anti-GAPDH, anti-phospho NfKB (Ser-536), and anti-NfKB antibodies were purchased from Cell Signalling Technology (Beverly, MA, USA). Anti-TMPRSS2 and anti-Flotillin-1 were purchased from Santa Cruz Biotechnology (Dallas, TX, USA). Anti-ACE2 antibody and the goat anti-rabbit secondary antibody and goat anti-mouse secondary antibody were purchased from Abcam (Cambridge, UK).

### 2.3. Cell Viability Assay

Cell viability was evaluated through colorimetric MTT metabolic activity assay as described in Proto, 2022 [26]. Briefly, cells were seeded into 96-well plates at a density of 5–8 × 10^3^ cells/well and treated in triplicate with Atorvastatin, Pravastatin (ranging from 1 to 20 µM), or vehicle (DMSO) as control for 24 or 48 h. At the end of treatments, MTT stock solution (5 mg/mL in PBS, Sigma, Darmstadt, Germany) was added to each well and incubated for 4 h at 37 °C in humidified CO_2_, then the medium was removed and the formazan crystals were solubilized with DMSO. MTT conversion to formazan by metabolically viable cells was monitored by spectrophotometer at an optical density of 540 nm. Each data point represents the average of at least three separate experiments in triplicate.

### 2.4. Western Blot Analysis

Western blot analysis was performed as previously described [27]. Total protein extracts were obtained by lysing cultured cells with ice-cold RIPA buffer (50 mM Tris–HCl pH 8.0, 150 mM NaCl, 1% Nonidet P-40) supplemented with protease and phosphatase inhibitors (Sigma). Protein concentration was determined through the Bradford method using bovine serum albumin as standard. 10–30 μg of proteins were loaded and subjected to 8–12% SDS-PAGE, under reducing conditions. Gels were electroblotted into nitrocellulose membranes that were probed with the primary antibodies. Membranes were incubated with an enhanced chemiluminescence (ECL) reagent solution (GE Healthcare, Hilden, Germany) and exposed to X-ray film (Santa Cruz, CA, USA) or to Amersham Imager 600 (GE Healthcare). Immunoreactive bands density was quantified with ImageLab v4.0 analysis software (Bio-Rad, Hercules, CA, USA) or with TotalLab Quant v2.2 software (TotalLab Ltd., St. Helens, United Kingdom).

### 2.5. Plasma Membrane-Derived Lipid Rafts Isolation

Lipid rafts from plasma membranes were obtained using the Minute™ Plasma Membrane-Derived Lipid Raft Isolation Kit (Invent biotechnologies) following the manufacturer’s instructions. Briefly, about 30 × 10^6^ cells were plated and treated with ATS, PVS, or vehicle for 24 h or 48 h. At the end, the cells were harvested, washed with ice-cold PBS, and centrifuged. The whole pellet obtained has been incubated in ice with the first buffer (Buffer A, containing phosphatase and protease inhibitors), followed by centrifugation through a spin-column system. The pellet obtained, containing nuclei and large cell debris, has been labeled as the “non-lipid rafts” fraction. The supernatant containing plasma membrane fraction (larger PM vesicles) has been centrifuged and treated with a non-ionic detergent containing buffer, followed by isolation of detergent-resistant fraction (white/grey-colored lipid raft) by flotation centrifugation. After aqueous phase removal, the highly enriched plasma membrane-derived lipid rafts have been labeled as the “lipid rafts” fraction. Both of the fractions obtained have been lysed in RIPA buffer.

The protein content from the lipid rafts fraction and the complementary fraction (Non-lipid rafts fraction) was determined using Bradford assay. Proteins were separated with 10% SDS-PAGE and detected by Western blot analysis.

### 2.6. Confocal Microscopy

Immunofluorescence staining was conducted as described in Fiore, 2018 [28]. A549-hACE2 and CaCO2 cells were grown in adhesion conditions on slides in 24-well plates. After treatment, cells were fixed in paraformaldehyde (PFA, 3.7% *v*/*v* in PBS) for 15 min, then washed and permeabilized in Tryton X-100 (0.1% *v*/*v* in PBS) for 10 min. Then, cells were blocked with 4% Bovine Serum Albumin (BSA) for 1 h at room temperature and incubated with anti-ACE2 and Flotillin-1 primary antibodies for 1 h at room temperature. Immunofluorescence staining was obtained by incubating for 1 h with Alexa Fluor^®^ 488 (FITC-conjugated) and Alexa Fluor^®^ 594 (Texas red-conjugated) secondary antibodies. Nuclei were stained with DAPI (4′,6-Diamidine-2′-phenylindole dihydrochloride) dye (Thermo Fisher Scientific). The slides were mounted using Mowiol mounting medium. Samples were vertically scanned through the Leica SP8 confocal microscope (Leica Microsystems CMS Gmbh; Mannheim, Germany) with a total depth of 10 μm and a Plan-Apochromat oil-immersion objective (magnification 63 × 1.7; 1.40 NA). A total of 20 z-line scans with a step distance of 0.5 μm were collected.

### 2.7. Analysis of Cytokines and Chemokines Profiles

Detection of IL6 levels in the culture media of A549-hACE2 cells treated with ATS and PVS alone or after the LPS stimulus was performed using Human IL6 Quantikine ELISA kit (R&D System), according to manufacturer’s instructions.

A Human Cytokine Antibody Array Membrane (Abcam) was used according to manufacturer’s instructions to analyse the supernatant from LPS-stimulated A549-hACE2 and CaCO2 cells treated with statins. Densitometric analysis of the membrane spots was obtained through TotalLab Quant v2.2 software (TotalLab Ltd.) and array data normalization was performed according to manufacturer’s instructions.

### 2.8. Statistical Analysis

Data obtained from multiple experiments were calculated as means ± SD and analysed for statistical significance by using the two-tailed Student t-test. All data shown are representative of at least three independent experiments performed in triplicate. Values of *p* < 0.05 were considered statistically significant.

## 3. Results

### 3.1. Statins Modulate ACE2 Receptor and TMPRSS2 Expression

Aiming to analyse the toxicity of statins, we examined the effect of ATS and PVS, lipophilic and hydrophilic statins, respectively, on cell viability in different cell lines expressing different levels of ACE2 receptor. For the range of concentration here examined (1 µM–20 µM), both ATS and PVS have shown a safe profile, without toxicity, in both A549-hACE2, overexpressing ACE2 receptor, thus highly permissive to SARS-CoV-2 infection and CaCO2 cell lines, constitutively expressing normal ACE2 receptor levels (Supplementary Figure S1A,B). In normal human lung fibroblast MRC5 cells, resistant to SARS-CoV-2 due to the low expression of ACE2 receptor [29], only ATS showed a significant cytotoxic effect, particularly pronounced at the highest concentrations (10 µM and 20 µM) (Supplementary Figure S1C).

**Figure 1:**
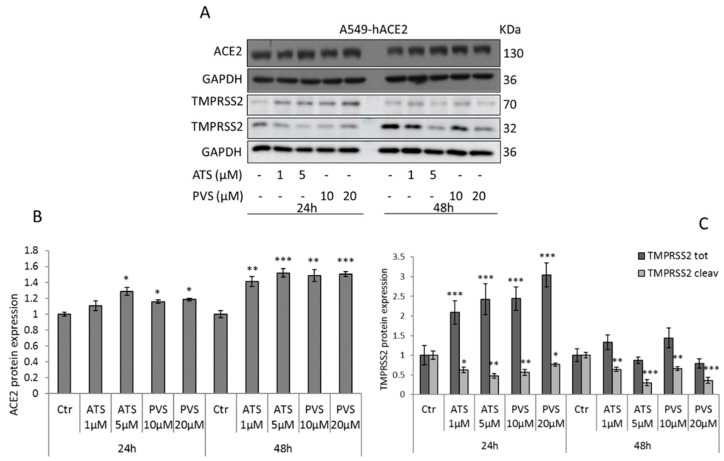
Representative Western blot and densitometric analyses of (**A**,**B**) ACE2 receptor and (**A**,**C**) TMPRSS2 (total and cleaved forms) expression in A549-hACE2 cells treated with ATS or PVS as indicated. GAPDH was used as loading control. Data are expressed as mean ± SD of at least three independent experiments. * *p* < 0.05, ** *p* < 0.01, *** *p* < 0.005 vs. control.

We next evaluated the effect of the compounds on ACE2 receptor and TMPRSS2 expression, in the highly permissive A549-hACE2 cells. As expected, after 24 h of treatment both ATS 5 µM and PVS 10–20 µM upregulate ACE2 receptor protein expression, but the strongest effect has been obtained after 48 h of treatment, where the compounds induce a strong upregulation (Figure 1A,B). After the binding with ACE2 receptor on host cells, SARS-CoV-2 spike proteins are activated by host cell protease TMPRSS2, responsible for S protein priming. The TMPRSS2-mediated cleavage at the S1/S2 site is required for viral entry into human lung cells [2,30]. We then analyzed the effect of the two statins on TMPRSS2 expression. After 24 h of treatment with both ATS and PVS, the expression of TMPRSS2 results markedly increased. However, prolonged time of treatment (48 h), with the highest concentrations of ATS (5 µM) and PVS (20 µM), produced a slight but not significant decrease of TMPRSS2 (Figure 1A,C). Interestingly, Western blot analysis allows us to detect an additional band of about 32 kDa which, based on the literature, corresponds to serine protease catalytic domain, directly involved in cell surface protein interaction, which is a product of auto-cleavage [31,32,33]. In this model, the cleaved domain of TMPRSS2 results in significant downregulation starting from 24 h of treatments with both the compounds and prolonged after 48 h in a dose-dependent manner (Figure 1A,C).

### 3.2. Statins Counteract the Effect of Induced Inflammation

**Figure 2:**
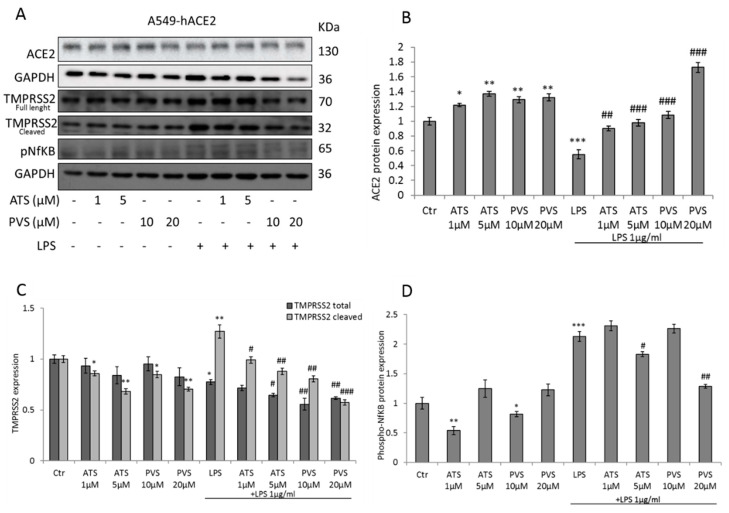
Representative Western blot and densitometric analyses of (**A**,**B**) ACE2 receptor, (**A**,**C**) TMPRSS2 (total and cleaved forms) and (**A**,**D**) phosphorylated-NfKB expression in A549-hACE2 cells treated with ATS and PVS, alone or after stimulation with LPS as indicated. GAPDH was used as loading control. Data are expressed as mean ± SD of at least three independent experiments. * *p* < 0.05, ** *p* < 0.01, *** *p* < 0.005 vs. control. # *p* < 0.05, ## *p* < 0.01, ### *p* < 0.005 vs. LPS alone.

To investigate the dynamic of statins during an inflammatory state, such as those induced by SARS-CoV-2 infection, we analyzed the expression of the key players involved in COVID-19, after LPS-induced inflammation, in vitro. In A549-hACE2 cells, as expected, LPS stimulus decreases the expression of ACE2 receptor. Pre-treatment for 48 h with ATS and PVS reverts the effect of LPS stimulus, inducing the re-expression of ACE2 receptor in a dose-dependent manner (Figure 2A,B). As previously described, prolonged treatment with statins downregulates both the full length and cleaved domain of TMPRSS2 in a dose-dependent manner. Despite LPS treatment reducing the full-length TMPRSS2, its cleaved domain results strongly increased. Interestingly, both ATS and PVS revert the LPS-induced upregulation of TMPRSS2 in the cleaved form, but also the expression of the full-length protein, compared to LPS and statins alone, with a better effect at the highest concentration (ATS 5 µM and PVS 20 µM) (Figure 2A,C). One of the first events after SARS-CoV-2 infection is the activation of NfKB pathway [34]. We then investigated if statins regulate NfKB activation, alone or after induced inflammation. Surprisingly, in A549-hACE2, only low concentrations of ATS (1 µM) and PVS (10 µM) significantly inhibited NfKB phosphorylation. However, after LPS stimulus, only the high concentrations (ATS 5 µM and PVS 20 µM) were able to revert the LPS-induced NfKB phosphorylation (Figure 2A,D).

**Figure 3:**
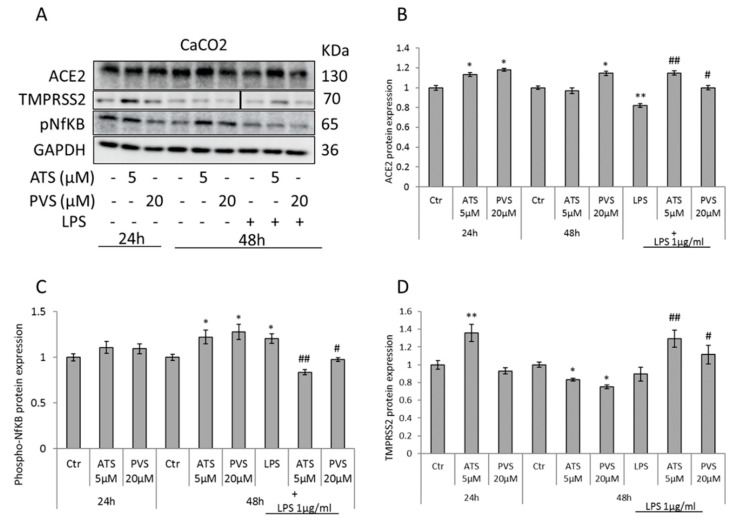
Representative Western blot and densitometric analyses of (**A**,**B**) ACE2 receptor, (**A**,**D**) total TMPRSS2 and (**A**,**C**) phosphorylated-NfKB expression in CaCO2 cells treated with ATS and PVS alone or after stimulation with LPS as indicated. GAPDH was used as loading control. Data are expressed as mean ± SD of at least three independent experiments. * *p* < 0.05, ** *p* < 0.01 vs. control. # *p* < 0.05, ## *p* < 0.01 vs. LPS alone.

To expand our analysis, we investigated the effects of statins in different in vitro models, expressing different levels of ACE2 receptor, using the concentrations found to be most efficient in the A549-hACE2 cells. In CaCO2 cells expressing normal levels of ACE2 receptor, after 24 h of treatment, PVS 20 µM and slightly ATS 5 µM induce protein expression of ACE2 receptor, but the effect is persistent after 48 h of treatment only with PVS. However, both the compounds seem to be able to revert the LPS-mediated reduction of ACE2 receptor, increasing its expression (Figure 3A,B). Surprisingly, neither ATS nor PVS used alone, at the highest concentrations, were able to reduce the levels of phosphorylated NfKB. However, as in A549-hACE2 cells, at the same concentrations ATS (5 µM) and PVS (20 µM) strongly revert the LPS- induced NfKB activation (Figure 3A,C). Of note, in CaCO2 cells, we unable to detect the TMPRSS2 cleaved band, corresponding to the cleaved serine protease domain. In line with other reports, it seems that CaCO2 cells express mostly the full-length protein and very low levels of autocleavage [35]. In this study, in CaCO2 cells both ATS and PVS were able to reduce TMPRSS2 at full length only after 48 h of treatment, but they are ineffective in reducing the protease expression in cells treated with a proinflammatory stimulus such as LPS (Figure 3A,D). Similar to what was observed in CaCO2, in MRC5 cells only PVS significantly increases the protein expression of ACE2 receptor, while ATS seemed to decrease it. Moreover, both of the compounds produced a significant up-regulation of ACE2 receptor, when combined with the LPS inflammatory stimulus. The expression of TMPRSS2 full length protein in MRC5 cells were not affected by the treatment with the two statins. However, the levels of the cleaved protein were almost completely abrogated after treatment with PVS, alone or with LPS (Supplementary Figure S2). Moreover, in MRC5 cells we observed a significant induction of active phosphorylated NfKB, after treatment with ATS 5 µM alone, which instead are reduced with both the compounds, after LPS induction. In particular, PVS reduces more than 50% of the levels of phospho-NfKB induced by LPS (Supplementary Figure S2).

Other authors reported the ATS-mediated activation of NfKB at highest concentration. They found a negative correlation between isoprenoid levels and NFkB activation, suggesting that inhibition of isoprenylated products mediated by statins, in some cells, may upregulate NfKB activity [36].

### 3.3. Effect of Statins on Chemokine/Cytokine Profile

**Figure 4:**
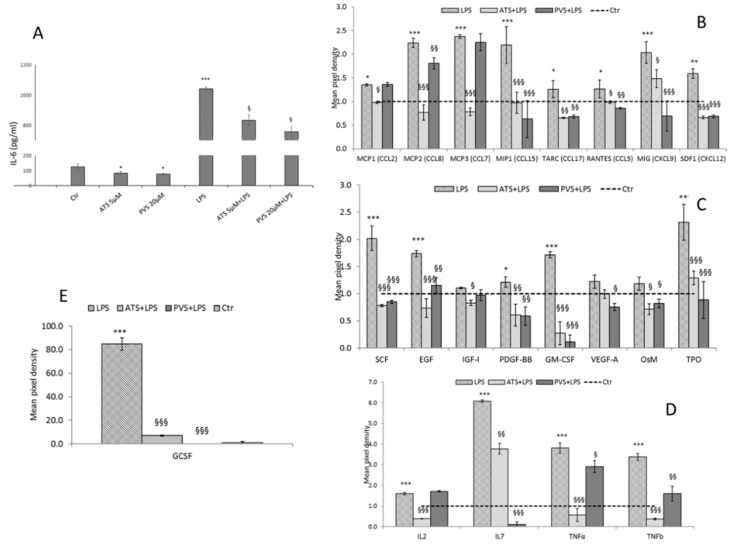
IL6 secretion in culture medium from A549-hACE2 cells treated with ATS and PVS alone or after stimulation with LPS, measured by ELISA (**A**). Effect of ATS and PVS on LPS-induced cytokines secretion in culture media from A549-hACE2 cells. Histograms represent densitometric analyses of the membrane spots on the antibody array (**B**–**E**). Data are expressed as mean ± SD of at least three independent experiments. * *p* < 0.05, ** *p* < 0.01, *** *p* < 0.005 vs. control. § *p* < 0.005, §§ *p* < 0.001, §§§ *p* < 0.005 vs. LPS.

Based on the results obtained, we quantified the amount of IL6 secreted in culture medium after treatment with the two compounds alone or after the LPS stimulus. In A549-hACE2 cells, after 48 h of treatment in basal conditions, both ATS and PVS reduce IL6 secretion; however, they do not reach statistical significance. As expected, LPS-induced inflammation causes a strong upregulation of IL6 secretion. Only PVS significantly reduces the LPS-induced IL6 secretion, while ATS produces a slight but not significant reduction (Figure 4A).

Using an antibody array membrane, we next analyzed the effect of lipophilic and hydrophilic statins, ATS and PVS, on a panel of 42 cytokines and chemokines typically associated with COVID-19 cytokine storm. Among the targets analyzed, in A549-hACE2 permissive cells, treated with ATS 5 µM or PVS 20 µM for 48 h and stimulated with LPS, we found that, with different trends, both statins significantly reduced the LPS-induced secretion of MCP2 (CCL8), MIP1 (CCL15), TARC (CCL17), RANTES (CCL5), MIG (CXCL9), SDF1 (CXCL12), SCF, EGF, PDGF-BB, Oncostatin (OsM), Thrombopoietin (TPO), IL7, TNFα, and TNF*β*. Interestingly, the levels of LPS-induced secretion of GCSF and GM-CSF were completely abrogated in ATS- and PVS-treated cells. Only ATS reduces the secretion of MCP1 (CCL2), MCP3 (CCL7), IGF-I, and IL2, while PVS significantly reduces the LPS-induced secretion of VEGF-A (Figure 4B–E). In CaCO2 cells, expressing normal ACE2 receptor levels, we found ATS more effective in the modulation of the secreted targets. In particular, both ATS and PVS significantly downregulated the LPS-induced secretion of ENA-78 (CXCL5), GCSF, IL4, IL5, and IL7 and caused a strong abrogation of IL6 secretion. However, ATS, but not PVS, reduces the levels of secreted MCP1, MIP1, MIG, GRO (CXCL1), MDC (CCL22), MCSF, Angiogenin, IL1*β,* and IL8. Interestingly, in CaCO2 cells, ATS also significantly reduces the levels of secreted IFNγ (Supplementary Figure S3). The data obtained confirm and sustain the efficacy of statins in counteracting or preventing the inflammatory status typically triggered by SARS-CoV-2 infection.

### 3.4. Hydrophilic and Lipophilic Statins Differentially Affect ACE2 Receptor Localization in Lipid Rafts

**Figure 5:**
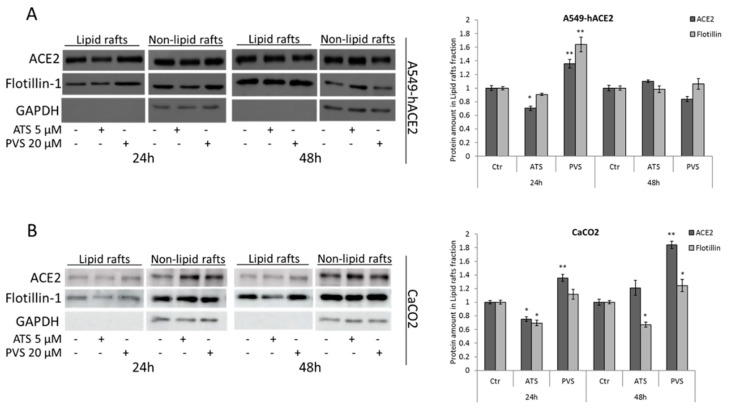
Representative Western blot analyses of ACE2 receptor and Flotillin-1 amount in lipid rafts and the complementary non-lipid rafts fractions, isolated from (**A**) A549-hACE2 and (**B**) CaCO2 cells, treated with ATS or PVS as indicated. Histograms on the right represent relative densitometric analyses of ACE2 receptor and Flotillin-1 in lipid raft fraction. GAPDH was used as loading control for the non-lipid rafts fraction. Data are expressed as mean ± SD of at least three independent experiments. * *p* < 0.05, ** *p* < 0.01 vs. control.

Our data demonstrated that the statin-mediated upregulation of ACE2 receptor is accompanied by the reduction of many cytokines and chemokines strongly involved in COVID-19, in agreement with their anti-inflammatory effect. The key used by SARS-CoV-2 to enter through the host cell membrane is ACE2 receptor, and its statin-mediated upregulation has raised considerable concern about the potential use of these compounds in COVID-19 infection. Aiming to dissect the dynamic of ACE2 receptor after treatment with statins, we isolated plasma membrane-derived lipid rafts, after treatment for 24 h or 48 h with ATS 5 µM or PVS 20 µM. We previously showed that in A549-hACE2 cells, at these concentrations, both ATS and PVS significantly increase ACE2 receptor expression (Figure 1). Nevertheless, as shown in Figure 5, the general increase of ACE2 receptor induced by statins after 24 h and 48 h of treatment is not verified in isolated lipid rafts. In overexpressing A549-hACE2 cells, ATS 5 µM does not induce expression of ACE2 receptor in the lipid rafts fraction. Rather, after 24 h of treatment we noticed a slight decrease in its lipid rafts localization, which coincides with a slight reduction of Flotillin-1 in fractionated lipid rafts. Instead, after 24 h of PVS 20 µM, the amount of ACE2 receptor also increases in the lipid rafts compartment, parallel with the increase of Flotillin-1 amount, but tends to decrease after 48 h (Figure 5A). Plasma membrane-derived lipid rafts have been also isolated from CaCO2 cells, where the lipophilic ATS produces the same effect observed in overexpressing cells, with the reduction of both ACE2 receptor and Flotillin-1 amounts in the lipid rafts fraction after 24 h of treatment. The reduction of Flotillin-1 also appears persistent and more evident after 48 h. However, accordingly to what is observed in A549-hACE2 cells, in CaCO2 the hydrophilic PVS increases in time-dependent manner the amount of ACE2 receptor in lipid rafts, accompanied with a slight increase in Flotillin-1 (Figure 5B). These data confirm the role of some statins in affecting lipid rafts composition but, on the other hand, prompt us to hypothesize that lipophilicity could affect this ability, since only ATS is able to produce lipid rafts perturbation and to affect ACE2 receptor localization within them.

**Figure 6:**
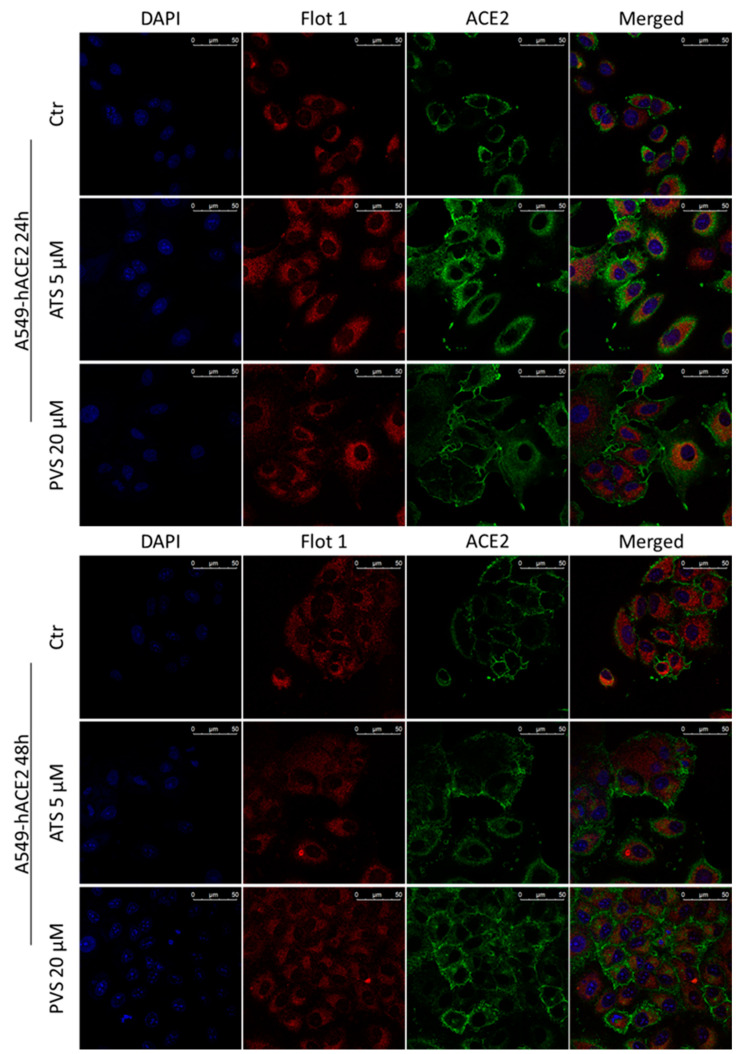
Representative confocal microscopy images of Flotillin-1 (red) and ACE2 receptor (green) in A549-hACE2 cells treated with ATS or PVS at indicated doses and time points. Nuclei were stained with DAPI (blue fluorescence). Scale bar 50 µm.

**Figure 7:**
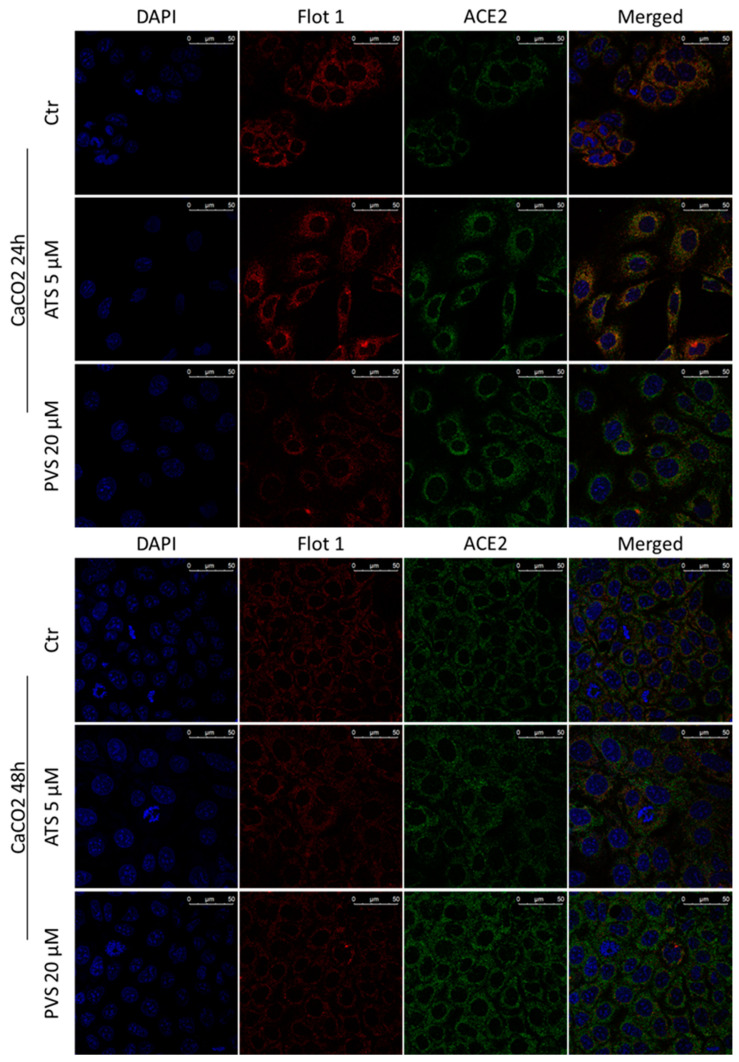
Representative confocal microscopy images of Flotillin-1 (red) and ACE2 receptor (green) in CaCO2 cells treated with ATS or PVS at indicated doses and time points. Nuclei were stained with DAPI (blue fluorescence). Scale bar 50 µm.

Confocal microscopy analysis (Figure 6 and Figure 7) substantially confirmed the increase in total ACE2 receptor expression in both cell lines analyzed, showing that its localization remains mainly intra-cytoplasm. The expression of total Flotillin-1 is coherent with the amount observed in the non-lipid rafts fraction showed in Figure 5.

## 4. Discussion

Statins are widely prescribed lipid-lowering drugs known for their pleiotropic effects, including anti-inflammatory, immunomodulatory, and anticoagulant properties [37]. As debated during the SARS-CoV-2 pandemic, the use of statins and their role in COVID-19 infection raised controversial opinions mainly sustained by their ability to induce ACE2 receptor expression and their adverse effects in some clinical conditions [16,17]. An impressive number of clinical studies attempted to clarify the safety, the therapeutic potential, and the impact of statins in clinical outcomes in COVID-19 patients [18,19,20,21,38]. However, since statins are drugs prescribed for patients with cardiovascular complications that constitute predisposing factors for SARS-CoV-2 infection, the results from these observational studies are often controversial due to multiple confounding factors and clinical bias influencing data interpretation [39]. Despite this, the overall conclusion emerging so far from several meta-analyses is that statins used in COVID-19 patients is associated with improved clinical outcomes and reduced Intensive Care Unit (ICU) admission, mechanical intubation, ARDS, and in-hospital death [20,40,41,42]. The studies included in these meta-analyses and emerging observational studies [43,44] strongly suggest that statin therapy exerts a protective effect against complications from COVID-19, preventing the worst prognosis. These effects are probably related to the immunomodulatory and anti-inflammatory properties of statins, but it has been also proposed that there is a possible direct inhibitory effect on the key steps of the virus life cycle, including internalization, replication, and virion release [13,39]. However, to date, few in vitro reports deciphered the exact molecular and cellular events underlying the protective effect of statins against infectious diseases such as COVID-19. In this work we tried to elucidate the effect of statins on specific traits associated with COVID-19 pathogenesis and course.

Aiming to understand the host genetic dependence, Danilosky and colleagues identified the genes required for SARS-CoV-2 infection. Notably, in CaCO2 and A549 ACE2-iperexpressing cells, they identified a group of genes involved in the cholesterol biosynthesis pathway and, intriguingly, strongly involved in SARS-CoV-2 infection. Among others they identified Rab7, a small-GTPase whose activity depends on post-translational prenylation, highlighting that it is involved in regulation of ACE2 receptor cell surface expression. In particular, loss of Rab7 causes an intracytoplasmic accumulation of ACE2 and, as a consequence, the sequestering of ACE2 receptor inside the cells translates into viral entry reduction [45]. Our results demonstrated that the global increase of ACE2 receptor expression mediated by statins does not necessarily coincide with its functional localization in lipid rafts domains, particularly after treatments with the lipophilic statins ATS. Moreover, in agreement with their role in perturbation of cholesterol contents and membrane fluidity, we demonstrated that ATS efficiently reduces the amount of Flotillin-1 in the lipid rafts fraction, suggesting that the displacement of ACE2 receptor from these domains is probably due to disruption of lipid rafts integrity. These data are coherent with a recent report highlighting that simvastatin, another lipophilic statin, also displaces ACE2 receptor from the cholesterol-enriched domains [24]. As observed, at the doses and times analyzed in our models, the hydrophilic statin PVS failed to interfere with lipid rafts composition and ACE2 receptor displacement. We hypothesize that this difference with ATS could be due to their different lipophilicities. This latter parameter is associated with the ability of statins to diffuse in the cell membranes. Passive diffusion across the plasma membrane is the preferential route used by lipophilic statins. However, at high doses and prolonged exposure, hydrophilic statins penetrate via both passive and active transport [46]. Hence, additional experiments are required to clarify the influence of the lipophilic features of statins in their ability to counteract viral mechanisms, also taking into account their pharmacokinetics, which could potentially explain the discrepancies and variabilities observed thus far in meta-analyses and clinical studies.

After its binding with ACE2 receptor and internalization in the host cells, SARS-CoV-2 induces a downregulation of ACE2 receptor that translates into an imbalance of its catalytic activity. It is clear that the downregulation of ACE2 receptor causes a perturbation in the renin angiotensin system (RAS) that exacerbates the COVID-19 pathogenesis [47,48]. Here we demonstrated that, in all the in vitro models analyzed, statins significantly counteracted the downregulation of ACE2 receptor after the LPS-induced inflammatory stimulus, re-inducing its expression. Moreover, the increase of ACE2 receptor was accompanied with a clear downregulation of TMPRSS2 serine protease domain, in iper-expressing cells A549-hACE2 cells. Interestingly, the increase of ACE2 receptor correlates with the inhibition of LPS-induced NfKB activation mediated by both ATS and PVS, with the exception of MRC5 cells, where only PVS reduced the phosphorylation of NfKB.

Our data show a contradictory result particularly evident in A549-hACE2 cells: When used in basal conditions, without an inflammatory stimulus, low doses of statins efficiently inhibit NfKB activation, but are insufficient to counteract the LPS stimulus. On the contrary, high concentrations of statins fail to inhibit NfKB activation in basal condition, but are clearly efficient in reverting the LPS effect. Noteworthy, through inhibition of HMGCoAR, statins prevent prenylation of a wide range of proteins, such as small GTPase, involved in signal transduction. In agreement with our findings, Arefieva and colleagues demonstrated that increasing concentrations of ATS induce the activation of NfKB, and this increase was inversely correlated with the reduction in FPP (farnesyl pyrophosphate) and GPP (geranyl pyrophosphate) mediated by the mevalonate pathway inhibitor, suggesting that isoprenoids may exert an inhibitory effect on NfKB activity [46]. Coherent with these observations, in a recent population-based cohort study including two million adult statin users, it has been highlighted that low-and moderate-intensity statins showed a lower risk of hospitalization and in-hospital mortality from COVID-19, whereas high-intensity statins did not [44].

Severity of COVID-19 is dictated by the failure of the immune response, which allows cytokine storm, ARDS, and organ damage. However, it is widely accepted that co-infections and pre-existing disease, such as cardiovascular disease, predispose to severe outcomes owing to their pre-hyperinflammatory status. In this frame, statins, the most prescribed lipid-lowering drugs for the prevention of cardiovascular complications, display a promising potential also due to their anti-inflammatory properties, and this constitutes a great and appealing advantage for the treatment of many diseases, including COVID-19. In a recent report, it has been demonstrated that simvastatin mitigates SARS-CoV-2-induced pro-inflammatory response in lung epithelial cells and immune cells (neutrophils and monocytes) [24]. Interestingly, Fluvastatin, another lipophilic statin, was also found to decrease the innate immune response in uninfected cells, enforcing the role of statins as immune-modulators in respiratory epithelia [25].

Multiple immune cells are involved in the pathogenesis of COVID-19, including monocytes, macrophages, dendritic cells, NK cells, T and B lymphocytes, that sustain the impressive and uncontrolled immune response induced by SARS-CoV-2. Cytokine storm implies an iper-production of inflammatory mediators, and specific chemokine signatures may be associated with different disease stages [4,6,25,49].

Here we characterized the effects of both lipophilic and hydrophilic statins on cytokine patterns in cells highly responsive to SARS-CoV-2, providing interesting data to better understand the protective role of these drugs against COVID-19. The limitations of this study are (i) the use of LPS as a stimulus to simulate SARS-CoV-2 infection, which could certainly involve a slightly different response, and (ii) the use of only one lipophilic (ATS) and one hydrophilic (PVS) statin, which certainly prevents full conclusions. Despite this, we found that, with different trends, both the compounds efficiently reduce the secretion of several factors correlated with SARS-CoV-2 infection. We noticed that, in our conditions, ATS shows the strongest effect in reducing the LPS-induced secretion of the majority of the examined cytokines, leading us to speculate that the observed differences could be due to lipophilicity. Overall, statins inhibit the release of cytokines and factors involved in the different stages of COVID-19 disease, but in particular of those produced in mild/severe disease (e.g., RANTES, MIG, SDF1, MCP1/2/3, MIP1, TNFα, IL2, IL7, and others) [4,49]. Particular attention must be paid to the inhibition of factors such as IL6, GCSF, GMCSF, and TNFα that are associated with highly severe disease and ICU admission of COVID-19 patients [4]. Of note, it has been reported that SARS-CoV-2 infection triggers growth factor receptor signaling and that its pharmacological inhibition prevents infection [50]. Coherently with their protective role, here we showed that both the statins can reduce the release of several growth factors, including SCF, EGF, IGF, PDGF-BB, VEGF, MCSF, GCSF, and GM-CSF.

In conclusion, the in vitro data emerging from our study provide useful additional information to dissect the mechanism underlying the potential protective effects of statin use in COVID-19. The results are coherent with the clinical reports published in the last two years that highlighted how statins, usually prescribed in patients with pre-existing cardiovascular disease and then frail patients with high risk of severe disease, protect patients from worse prognoses, ICU admissions, and death [41,42,43,44]. The understandings of statins’ molecular mechanisms in COVID-19 could have a relevant translational impact on disease management. In particular, knowing the response to treatment based on statin type, doses, and regimen can help predict which COVID-19 patients may benefit from therapy, as well as predict prognosis and outcomes, in order to plan patient-specific therapeutic protocols that are readily usable and cost-effective. Of course, additional studies are urgently needed to further understand how pharmacodynamic features, lipophilic features, doses, and time of treatment could substantially improve the outcome in COVID-19 patients.

## Supplementary Materials

The following supporting information can be downloaded at: https://www.mdpi.com/article/10.3390/biomedicines10092123/s1, Figure S1: Cytotoxic effect of statins in vitro; Figure S2: Statins effects in MRC5 cells; Figure S3: Effect of statins on cytokines secretion in CaCO2 cells.

## Abbreviations

EGF	Epidermal growth factor
ENA-78	Epithelial-neutrophil activating peptide
GCSF	granulocyte colony-stimulating factor
GM-CSF	Granulocyte-Macrophage Colony-Stimulating Factor
GRO	growth-regulated oncogene
IFN-γ	Interferon gamma
IGF-I	Insulin-like growth factor-1
IL	Interleukin
IP-10	Interferon gamma-induced protein 10
MCP1	Monocyte chemoattractant protein-1
MCP2	Monocyte chemoattractant protein-2
MCP3	Monocyte chemoattractant protein-3
MCSF	macrophage colony-stimulating factor
MDC	Macrophage-derived chemokine
MIG	Monokine induced by gamma
MIP1	Macrophage Inflammatory Protein-1
PDGF-BB	Platelet-Derived Growth Factor-BB
RANTES	Regulated upon Activation, Normal T Cell Expressed and Presumably Secreted
SCF	Stem cell factor
SDF1	stromal cell-derived factor 1
TARC	Thymus and activation-regulated chemokine
TGF	transforming growth factor
TNF*β*	Tumor necrosis factor-β
TNFα	Tumor necrosis factor-α
