# Supplementary material for: In Vitro Evidence of Statins’ Protective Role against COVID-19 Hallmarks Comptes Rendus
Source: Biomedicines. 2022 Aug 29;10(9):2123. doi: 10.3390/biomedicines10092123 (PMC9495908; doi:10.3390/biomedicines10092123)
Supplement: Supplementary file 1 [file biomedicines-10-02123-s001.zip › biomedicines-1878207-supplementary.pdf]

Supplementary Figure S1.

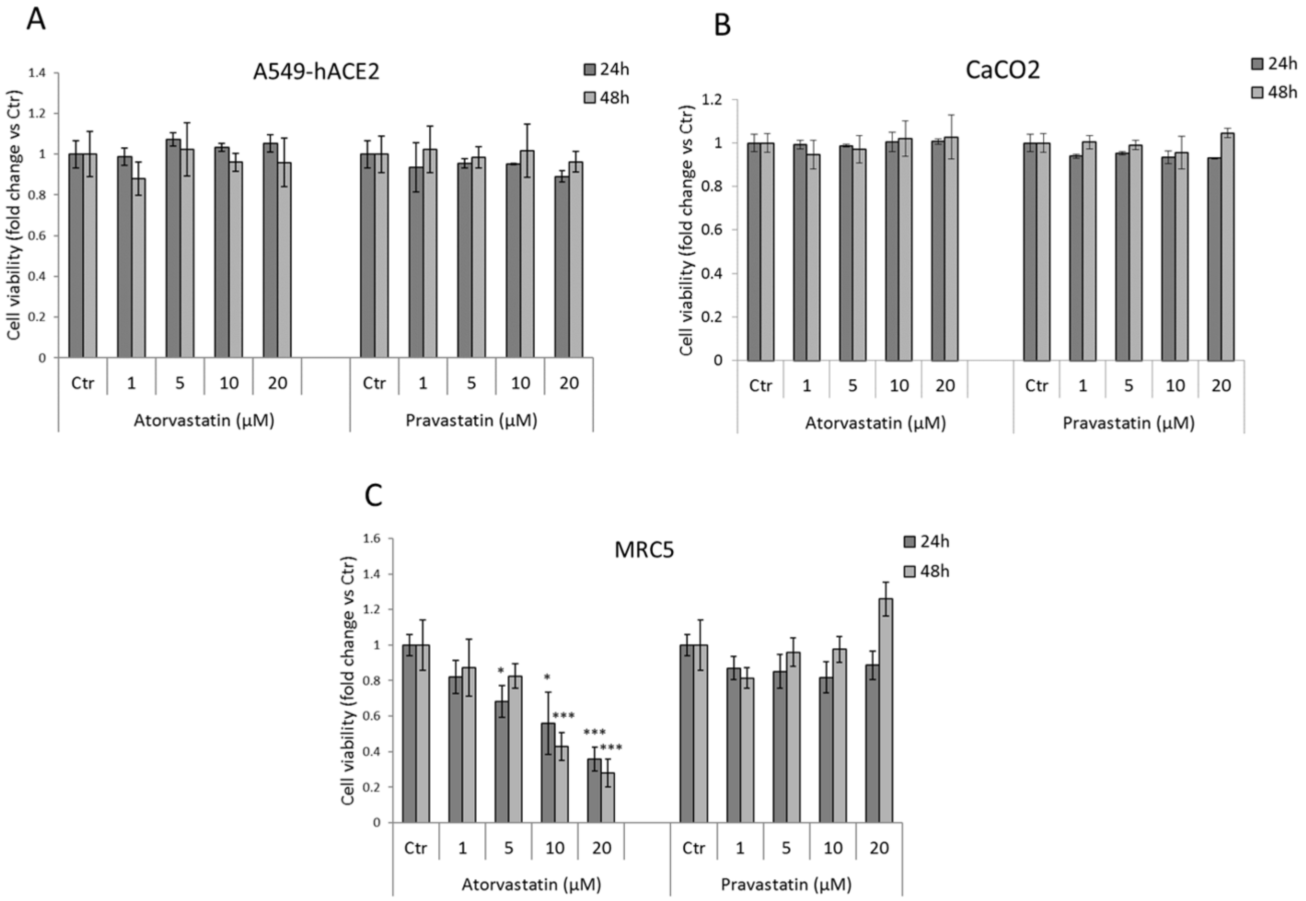

Supplementary Figure S1. Cytotoxic effect of statins *in vitro*. Cell viability MTT assay in (**a**) A549-hACE2, (**b**) CaCO2 and (**c**) MRC5 cells treated with ATS or PVS (1 – 20 μM) for 24-48h. Data are expressed as mean ± SD of at least three independent experiments. * $p < 0.05$, ** $p < 0.01$, *** $p < 0.005$ vs. control.

Supplementary Figure S2.

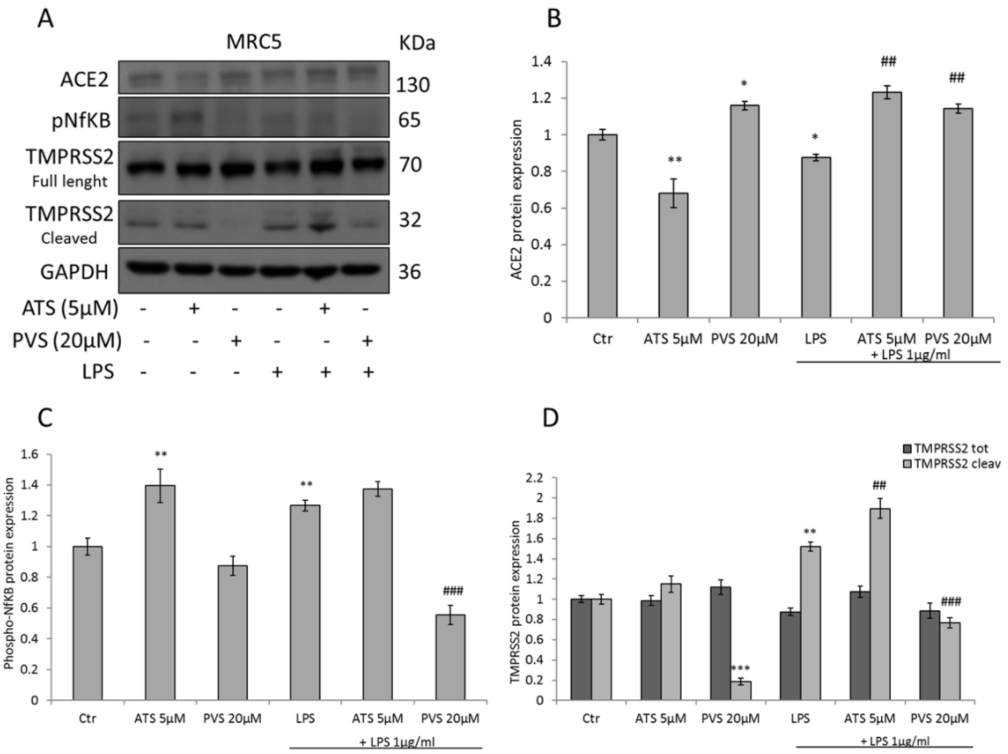

Supplementary Figure S2. Representative western blot and densitometric analysis of (**a, b**) ACE2 receptor, (**a, d**) TMPRSS2 (total and cleaved forms) and (**a, c**) phosphorylated-NfKB expression in MRC5 cells treated with ATS and PVS alone or after stimulation with LPS as indicated. GAPDH was used as loading control. Data are expressed as mean ± SD of at least three independent experiments. * $p < 0.05$, ** $p < 0.01$, *** $p < 0.005$ vs. control. ## $p < 0.01$, ### $p < 0.005$ vs. LPS alone.

Supplementary Figure S3.

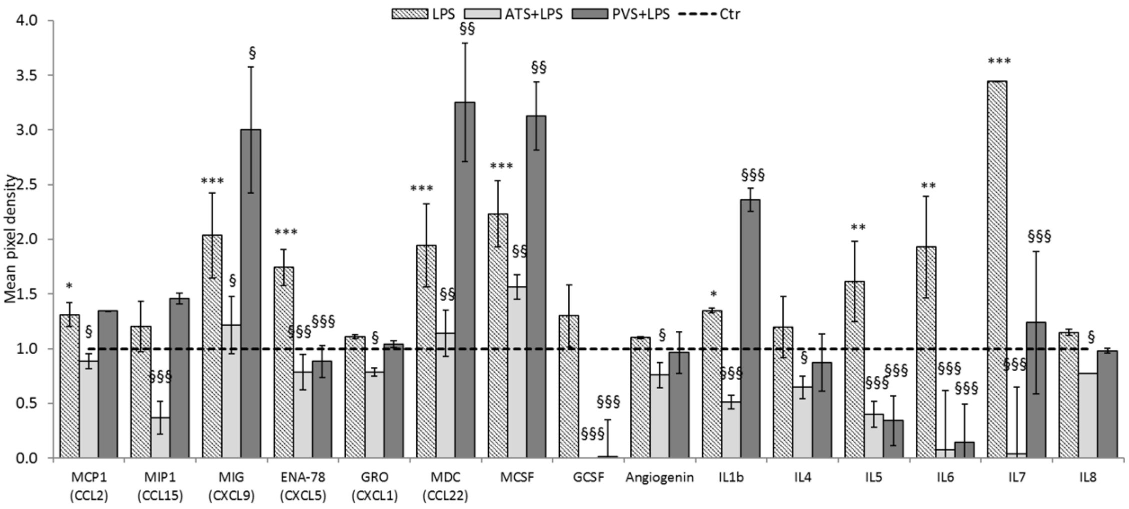

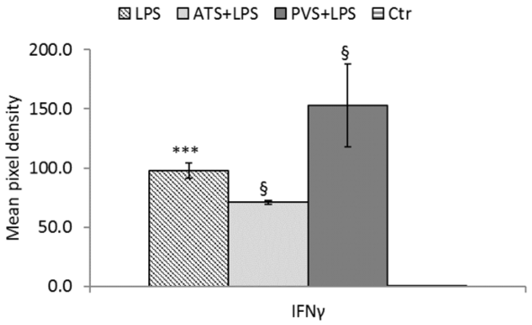

Supplementary Figure S3. Effect of ATS and PVS on LPS-induced cytokines secretion in culture medium from CaCO2 cells. Histograms represent densitometric analysis of the membrane spots on the antibody array. Data are expressed as mean ± SD of at least three independent experiments. * p < 0.05, ** p < 0.01, *** p < 0.005 vs. control. § < p 0.005, §§ p < 0.001, §§§ p < 0.005 vs. LPS.
